# Retention of HIV-Positive Adolescents in Care: A Quality Improvement Intervention in Mid-Western Uganda

**DOI:** 10.1155/2018/1524016

**Published:** 2018-05-06

**Authors:** Jonathan Izudi, John Mugenyi, Mary Mugabekazi, Benjamin Muwanika, Victor Tumukunde Spector, Andrew Katawera, Adeodata Kekitiinwa

**Affiliations:** ^1^Baylor College of Medicine Children's Foundation Uganda, Center of Clinical Excellence, Research Unit, P.O. Box 72052, Block 5 Mulago Hospital, Kampala, Uganda; ^2^Department of Community Health, Faculty of Medicine, Mbarara University of Science and Technology, P.O. Box 1410, Mbarara, Uganda; ^3^Clarke International University (Formerly International Health Sciences University), Institute of Public Health and Management, P.O. Box 7782, Kampala, Uganda; ^4^Ministry of Health Uganda, Kyenjojo District Local Government, Katooke Health Center III, Kyenjojo District, Uganda; ^5^Baylor College of Medicine Children's Foundation Uganda, Rwenzori Regional Office, P.O. Box 72052, Block 5 Mulago Hospital, Kampala, Uganda

## Abstract

**Background:**

Low retention of HIV-positive adolescents in care is a major problem across HIV programs. Approximately 70% of adolescents were nonretained in care at Katooke Health Center, Mid-Western Uganda. Consequently, a quality improvement (QI) project was started to increase retention from 29.3% in May 2016 to 90% in May 2017.

**Methods:**

In May 2016, we analyzed data for retention, prioritized gaps with theme-matrix selection, analyzed root causes with fishbone diagram, developed site-specific improvement changes and prioritized with countermeasures matrix, and implemented improvement changes with Plan-Do-Study-Act (PDSA). Identified root causes were missing follow-up strategy, stigma and discrimination, difficult health facility access, and missing scheduled appointments. Interventions tested included generating list of adolescents who missed scheduled appointments, use of mobile phone technology, and linkage of adolescents to nearest health facilities (PDSA 1), Adolescent Only Clinic (PDSA 2), and monthly meetings to address care and treatment challenges (PDSA 3).

**Results:**

Retention increased from 17 (29.3%) in May 2016 to 60 (96.7%) in August 2016 and was maintained above 90% until May 2017 (with exception of February and May 2017 recording 100% retention levels).

**Conclusion:**

Context specific, integrated, adolescent-centered interventions implemented using QI significantly improved retention in Mid-Western Uganda.

## 1. Introduction

Besides the challenges of rapid physical, emotional, cognitive, and social changes, adolescents (10–19 years) have increased risk of poor health outcomes and acquisition of HIV (Human Immunodeficiency Virus) infection [1]. Currently, HIV associated morbidity and mortality and quality of life have remarkably improved with availability of life-saving ART (antiretroviral therapy) due to virologic suppression [2, 3]. However, AIDS (Acquired Immunodeficiency Syndrome) still kills many HIV-positive adolescents globally, mostly in Sub-Saharan Africa [4]. To prevent considerable HIV related morbidity and mortality, all HIV-positive adolescents must be put on antiretroviral therapy (ART) and should remain in care to achieve virologic suppression [4]. Thus, early entry and retention in HIV medical care increases the success of the test and treatment strategy, a policy guideline where all persons who test HIV positive are started on ART irrespective of their age, immune, and clinical status [1, 5]. Retention is thus a critical requirement for achieving favorable long-term clinical outcomes of HIV treatment [6]. Indeed, failure to keep medical appointments usually leads to poor treatment outcomes [7]. Retention of HIV-positive adolescents must accordingly be a top priority of HIV prevention, care, treatment, and support programs [8].

Nonetheless, retention remains a substantial challenge across several HIV-programs worldwide. One study conducted by the AIDS Support Organization (TASO) in Uganda showed that 65% of adolescents remained in care at the end of the fifth year [9]. Elsewhere, nonretention increased with the duration of ART and adolescent age, with older adolescents (15–19 years) having higher nonretention rate compared to younger adolescents aged 10–14 years [10]. At Katooke Health Center (study setting), Kyenjojo district in Mid-Western Uganda, substantial number of HIV-positive adolescents are not retained in care. Evidence from a three-month data analysis indicated suboptimal retention levels: 17 (29.3%), 23 (39.7%), and 20 (34.5%) in May, June, and July 2016, respectively.

This retention level was unacceptably lower than the Uganda Ministry of Health recommendation of at least 90% retention. The situation was further worsened by lack of site-specific measures to address the performance gap. Moreover with suboptimal retention, HIV-positive adolescents risk ART disruption, and this leads to poor adherence and suboptimal virologic nonsuppression. In the end, HIV associated morbidity and mortality, and poor quality of life emerges. Finally, the important goal of ART in preventing HIV transmission is lost. We therefore initiated a quality improvement (QI) project to raise the retention of HIV-positive adolescents in care from 29.3% in May 2016 to 90% in May 2017.

## 2. Methods

### 2.1. Study Design

We used a QI design to address low retention rates of HIV-positive adolescents at Katooke Health Center. QI is a science for improving quality of health services and for achieving the 90-90-90 HIV targets [1]. It requires routine use of data to improve processes and systems of health service delivery so as to meet patient/program needs, to measure current performance and compare it with standard performance [11], and to take deliberate efforts guided by causal analysis, intelligent direction, and skillful execution to address deficiencies [12].

### 2.2. Study Setting

Katooke Health Center is a government owned health facility located in Kyenjojo district, Mid-Western Uganda. It is located at latitudes 0°38′42.7′′ (0.6452°) in the north and longitudes 30°39′18.8′′ (30.6552°) in the east. The total catchment population of the health facility for the period July 2017 and June 2018 is 19, 2003 people, of whom 51.3% are females, while 35.0% are adolescents (10–19 years). The Health facility's HIV clinic was started in 2014 to offer comprehensive HIV care. As of March 2018, 1,355 people (60 of whom are HIV-positive adolescents) were cumulatively active in care.

The HIV clinic is run by six healthcare providers: a Clinical Officer, two Nurses, a Medical Records Assistant, a Dispenser, and a Midwife. The HIV clinic is accessible throughout the week, however. The Health Facility has a HIV QI Team that was formed by Baylor College of Medicine Children's Foundation (in short, Baylor Uganda) in 2010 with funding from CDC (Centers for Disease Control and Prevention) under PEPFAR (United States Presidents Emergency Plan for AIDS Relief). The mandate of the HIV QI Team is to identify and address gaps in HIV service delivery using QI approach.

### 2.3. Baseline Assessment

Baylor Uganda supported the HIV QI Team to retrospectively review HIV data for retention levels between May and July 2016. We found high retention level (95.7%) among children below 10 years, slightly better retention level (87.5%) among adults aged 20-years and above, and an unacceptably lower retention level (29.3%) among adolescents aged 10–19 years, in May 2016 (Table 1). Between June and July 2016, we started to rejuvenate the HIV QI Team through several approaches. First, we conducted a 1-hour Continuous Medical Education (CME) session on basic concepts of QI: introduction to QI, dimensions of quality, the quality grid, steps and principles of QI, data use in QI, tools used for problem identification, and data analysis in QI among others. The purpose of the CME session was to build or rebuild the capacity of healthcare providers in using QI approaches to address performance gaps. Second, we reconstituted the health facility QI Team with a member from each department and formed departmental QI teams to address performance gaps at departmental level. In our last mentorship and meeting in July 2016, we supported the HIV QI Team to start a QI project to address the low retention of HIV-positive adolescents in care. We followed four major steps per requirements of QI [13].

*Step 1: Problem Identification.* This involved the review and abstraction of data for baseline performance (Table 1). This was followed by use of a theme-matrix selection to prioritize identified gaps for improvement based on its urgency and impact on public health using rank scores of 1–5. In Table 1, a gap that was regarded by the HIV QI Team as urgent and with huge public health impact received the highest ranked score of five, while those that were considered less urgent to be solved and with low public health impact received the lowest ranked score of one. On the other hand, gaps that were considered intermediate received between two and four ranked scores. The overall score was a product of the urgency and impact scores. Consequently, the overall scores for retention of children below 10-years, adolescents (10–19 years), and adults (20 years and above) were 20, 25, and 22.5 respectively (Table 1). Since the retention of HIV-positive adolescents in care received the highest overall score, it was selected for improvement by the HIV QI Team.

**Figure 1:**
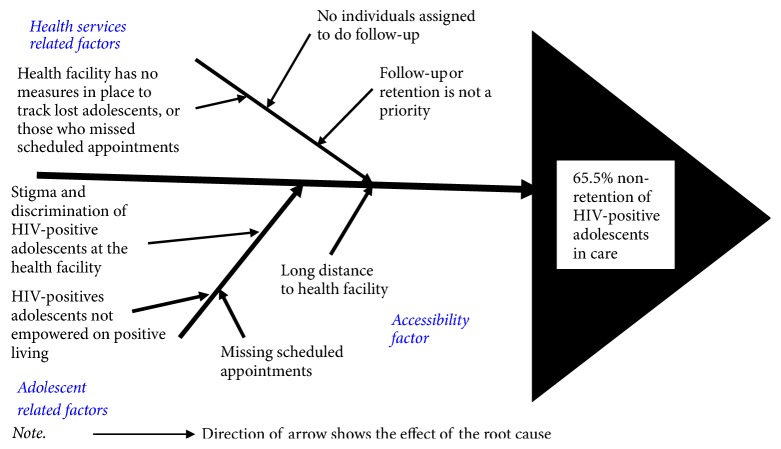
Fishbone tool for analysis of root cause of low retention of HIV-positive adolescents in care, Katooke HCIII, Kyenjojo district.

*Step 2: Root Cause Analysis.* We used the fishbone diagram to analyze the root causes of nonretention (65.5%) of HIV-positive adolescents in care (Figure 1). We adopted the 5-whys approach in determining potential root causes of nonretention. Each identified root cause was subjected to further questioning for a minimum of five times until a reasonable answer was obtained. This approach ensured an exhaustive analysis of all contextual root causes of low retention in an orderly manner [14]. We then grouped the root causes into three main categories: health services, adolescent, and accessibility related factors.

Under health services related root causes, the absence of onsite measures to fast-track HIV-positive adolescents who miss clinic appointments (not appearing in the HIV clinic for 7 days since scheduled appointment date) or who get lost (not appearing in the HIV clinic for 90 days since last clinic visit), the absence of an individual in the HIV clinic assigned to monitor retention and the lack of priority given to nonretention or follow-up of adolescents who miss scheduled appointments or who get lost emerged as subcauses. Second, adolescent related subcauses included stigma and discrimination during routine medical visits, inadequacies in empowerment to live positively, and missing scheduled appointments. Third, a long distance (more than 5 kilometers) to the health facility was the only single subcause attributed to accessibility factor (Figure 1).

**Figure 2:**
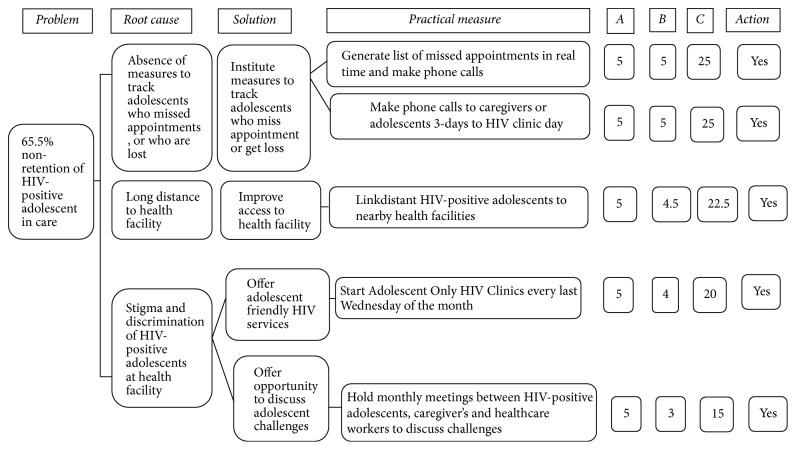
Countermeasures matrix showing prioritization of quality improvement changes for addressing low retention of HIV-positive adolescents in care, Katooke HC III, Kyenjojo district.* Note*. A: effectiveness score; B: feasibility score; C: product of A and B; action: yes = accepted for quality improvement, no = rejected for quality improvement.

*Step 3: Developing and Prioritizing Solutions.* We used the countermeasures matrix to develop improvement solutions and practical measures, which was thereafter ranked using scores of 1–5 for its effectiveness and feasibility. A high score of five was assigned to a practical measure that was deemed effective in addressing identified root cause and feasible to implement using local resources, and vice versa (Figure 2). The overall score was the product of effectiveness and feasibility scores [15].

Regarding the absence of measures to track adolescents who miss clinic appointments or who get lost, the practical measures included generating list of adolescents who missed scheduled HIV clinic appointments in real time (at the end of each clinic day) followed by a mobile phone call to the respective parents or legal guardians that scored 25. Second, a three-day prior mobile phone call to parents or legal guardians of HIV-positive adolescents before scheduled HIV clinic appointment date equally scored 25. For adolescents who travelled longer distance to reach the health facility (Katooke Health Center), the practical measure was linkage to a nearby health facility that scored 22.5 (Figure 2).

In tackling stigma and discrimination that HIV-positive adolescents face while at the HIV clinic, two practical measures emerged. First, staring an Adolescent Only Clinic (AOC) on every last Wednesday of the month scored 20. Second, holding monthly meetings with adolescents, their parents or legal guardians to discuss care and treatment challenges scored 15 (Figure 2).

*Step*  *4: Implementation of Improvement Changes.* The Plan-Do-Study-Act (PDSA) cycle, a series of steps for improving processes [16, 17]; to test and implement improvement changes [18, 19]; and to plan, execute, observe, and determine modifications needed during implementation of improvement changes [16] was used to implement the practical measures. In general, the PDSA cycle supported the easy implementation, monitoring, and evaluation of improvement changes during implementation [20]. In this QI intervention, the first PDSA cycle consisted of three interventions: (1) generating list of adolescents who missed scheduled clinic appointments in real time followed by mobile phone calls to the respective parents, or legal guardian, (2) making mobile phone calls to parents or legal guardians three-days prior to scheduled HIV clinic day, and (3) linking distant adolescents to nearby health facilities that offer HIV services. We used an appointment register/book to track adolescents who missed scheduled clinic appointments and an ART register to track adolescents who got lost. These three interventions were implemented simultaneously in August 2016. The second PDSA cycle consisted of starting an AOC in January 2017. During AOC days, HIV-positive adolescents received care and treatment from health workers trained in provision of adolescent friendly HIV services from 8.00 a.m. to 5.00 p.m. on every last Wednesday of the month. Adolescents also participated in various recreational activities like playing football and netball and watching educative films among others.

The third PDSA cycle consisted of monthly meetings with HIV-positive adolescents, their respective parents or legal guardians, and healthcare providers in February 2017. The creation of this meeting provided an avenue to discuss adolescent HIV treatment and care challenges in a holistic manner.

### 2.4. Statistical Analysis, Documentation, and Dissemination of Results

Retention was measured according to the Uganda Ministry of Health definition as the number of HIV-positive persons with at least one HIV clinic visit in a period of 90-days, expressed as percentage. The denominator was the number of HIV-positive persons in the review period. We excluded HIV-positive adolescents who transferred to other health facilities. A QI documentation journal that consisted of start and end dates of the QI project, an improvement objective, an indicator for the improvement objective, a problem statement for the objective, planned and tested improvement solutions (practical measures), a line graph with annotations, and a documentation of lessons learnt during the QI intervention was used to track and report our progress. On monthly basis, a QI meeting was held during which the documentation journal was updated with current retention levels and an explanation was provided with annotations. Also, the QI team members were updated on the progress of the project and improvement changes were modified, whenever needed.

### 2.5. Ethical Approval

Ethical approval was obtained from Baylor Uganda Research Ethics Committee in addition to a Memorandum of Understanding (MoU) signed between Baylor Uganda and Kyenjojo District Local Government Health Office.

## 3. Results

**Figure 3:**
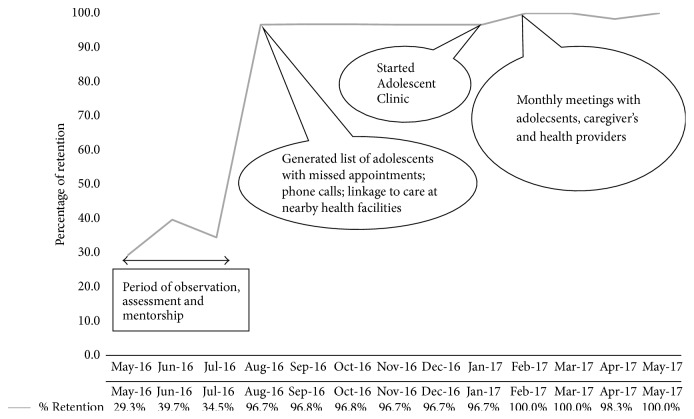
Line graph showing percentage of HIV-positive adolescents retained in care over time, Katooke HC III, Kyenjojo district.

Without interventions (Figure 3), 17 (29.3%) HIV-positive adolescents were retained in care in May 2016. However, 23 (39.7%) and 20 (34.5%) were retained in care in June and July 2016, respectively. Figure 3 shows that, with inception of QI interventions in July 2016, the number of HIV-positive adolescents retained in care rose from 20 (34.5%) in July 2016 to 60 (96.7%) in August 2016 and then to 62 (96.8%) in September 2016. This level of retention was maintained until October 2016. However, in August 2016, four new HIV-positive adolescents were enrolled in care at the health facility; thus, the total number of adolescents in care was 62. Retention then stagnated between October 2016 and November 2016 (62 (96.8%) versus 60 (96.7%), resp.) and this level of retention in November 2016 was maintained until January 2017. In February 2017, two of the adolescents transferred to other HIV providing health facility; thus, the total number of adolescents in care dropped to 60. However, retention improved from 60 (96.7%) in January 2017 to 60 (100.0%) in February 2017 and was sustained until March 2017. In April, one HIV-positive adolescent was enrolled in care and the number of adolescents rose to 61 and slight drop in retention occurred from 60 (100.0%) in March 2017 to 60 (98.3%) in April 2017. In May 2017, one adolescent transferred to another health facility, so the total number of adolescents in care dropped to 60, and all were retained in care. In a separate analysis, we found that 74.4% of all adolescents who were retained in care had accessed viral load monitoring and 81.9% attained virologic suppression (Supplementary Material).

## 4. Discussion

In this QI project, our objective was to increase the retention of HIV-positive adolescents in care at Katooke Health Center, Kyenjojo district, Mid-Western Uganda, through simple, evidence-derived but practical interventions. QI is a new concept in Uganda's Healthcare System because focus by Ministry of Health was largely on quantity of health services in the past [21, 22]. It was in 2010 when the first QI Strategic Framework was launched to mark nationwide use of QI approaches at various levels (national, regional, district, health subdistrict, hospitals and its departments, and health centers) [22] to address healthcare performance gaps. The current (second) Health Sector QI Framework and Strategic Plan covers the period 2015/2016 to 2019/2020, and the goal is to provide high quality health services and attain good quality of life and well-being at all levels of healthcare delivery in Uganda [21, 23]. It is important to note that quality of healthcare mediates the relationship between the six WHO (World Health Organization) building blocks of health systems strengthening (service delivery, health work force, health information, health financing, leadership and, medical products, vaccines, and technologies) and health outcomes (effectiveness, efficiency, responsiveness, and social and financial risk protection) [1, 22].

In this QI project, retention improved tremendously with our first set of interventions in which adolescents who missed scheduled HIV clinic appointments were tracked in real time using an appointment book and by making mobile phone calls to the respective parents or legal guardians, and by linking distant adolescents to nearby health facilities. In real time follow-up, we generated a list of HIV-positive adolescents who missed scheduled appointments at the end of the HIV clinic day using an appointment register. This enabled fast-tracking with mobile phone calls to respective parents or legal guardians to establish reasons for nonturn up and to reschedule an immediate visit in 3–5 days. In Uganda, the appointment register forms the main tracking tool for HIV-positive persons who miss scheduled visits across HIV clinics.

In addition, although the use of mobile phone technology in public health and medicine (mHealth) is still in its infancy stages in Uganda, mobile phone calls (when appropriate and feasible) and text messaging are recommended by the Ministry of Health to track HIV patients who miss scheduled appointments, or who get lost [1]. In adolescent HIV care, mHealth improves medication adherence and helps achieve viral suppression [24]. In fact, mHealth is proven to be feasible and acceptable in HIV care [24, 25] and can be applied to various categories of patients for reminder purposes. In a study on substance-dependent patients, prospective phone call reminders on initial scheduled appointments and follow-up phone calls to those who failed to show up improved the rate of treatment initiation [26].

Earlier, the use of mobile phone calls led to 60% retention of HIV-positive pregnant and lactating mothers and 75% retention of HIV-positive children at 1 year in Uganda [1]. Our intervention and results are therefore consistent with acceptable innovative approaches in HIV programming. However, it is important to note that mHealth comes at a cost which must be met by both the healthcare system and the patient. For the healthcare system, sufficient funds are needed for buying and installing a desktop phone at the health facility, maintaining, and repairing as well. In addition, sufficient airtime must be availed and healthcare workers trained in effective business communications skills. For the patient, the ability to purchase and maintain a functional handheld mobile phone is critical.

Our intervention may therefore not apply in settings where the above factors are a challenge. Lastly, with frequent power blackouts in developing countries, mHealth may in general face huge challenges. However, mHealth is an important adjunct intervention for improving retention. The implementation of mHealth is thus challenging in both developing and developed countries. For instance, in the United States, a study showed that the use of text messaging in HIV care was not feasible for several reasons: lack of either a cell phone or text messaging service; cost; comfort with text messaging; and privacy [7]. The same study pinpointed that text message reminders may be successful in improving clinic attendance in certain groups of patient as some barriers that retard its universal application must be tackled [7].

By linking distant adolescents to nearby health facilities that provide comprehensive HIV services, we removed direct and indirect costs associated with accessing health services from a distant health facility. This is consistent with earlier results of systematic review in the United States [27].

Still in a rural setting of the United States, good patient to provider relationships, family support, easy access to transport, organizational infrastructure of healthcare facility, and absence of HIV stigma within communities improved retention of HIV-positive women's retention in care [6]. When health facilities are distant (more than 5-kilometer radius), patients have to pay for motorized transport system (if able and willing) or must walk for longer hours to reach the health facility. This hence deters effective use of available and free HIV services. Past evidence indicates that patients who receive HIV care services at a health facility were less retained in care compared to patients who receive HIV care services at a community health post in Uganda [10].

Retention also significantly improved with the initiation of an AOC on every last Wednesday of the month. Adolescent friendly clinics are characterized by three main elements: the target population like pediatric, adolescent; the physical environment; and the social environment. Such clinics have favorable changes in their physical (space, entertainment, and educational materials) and social (staff training related to development, gender, and sexual orientation) environments that reduce HIV-infected adolescents' unique barriers to care. In addition, they integrate clinic designs and train staffs within their clinical program in order to meet the specialized needs of HIV-infected adolescents [28].

In establishing the AOC, a convenient date was unanimously agreed upon by adolescents, healthcare providers, and parents or legal guardians. We noted that the AOC provided a friendly environment for adolescents in that health education talks were led by adolescent peer leaders under the guidance of health workers, health care was provided by health workers who were trained in adolescent friendly HIV services, and adolescents participated in several recreational activities (singing; playing: netball, football, chase, and athletics; and watching educative movies). Consequently, the AOC became more acceptable, suitable, enjoyable, and interesting to all stakeholders. Importantly, health workers had ample time to concentrate on critical adolescent HIV care challenges such as adherence to ART, viral load monitoring, and psychosocial support. This intervention is consistent with the Uganda Ministry of Health recommendation of providing adolescent friendly HIV services so as to enhance retention [1]. Past study conducted in Kenya, Mozambique, Tanzania, and Rwanda showed that adolescent friendly services reduce attrition (nonretention) among youths, particularly after the initiation of ART [29]. Consistent with the present study, in the United Sates, the linkage of HIV-positive adolescents to care in 15 different clinics eliminated structural barriers that hindered retention [30].

Retention equally improved when monthly meetings between adolescents, health workers, and parents or legal guardians were introduced to tackle adolescent HIV care and treatment challenges. The meetings provided a golden opportunity to counsel and educate adolescents and parents or legal guardians on ART adherence, positive living, stigma and discrimination, socioeconomic empowerment, management of ART side effects, and importance of compliance to scheduled appointments, among others. Through this interaction, most adolescents were empowered to live positively and parents or legal guardians gained knowledge in tackling adolescent challenges. Indeed, for adolescents to take control over their own health and the determinants of health in general, empowerment is crucial [1]. According to a systematic review in the United States, retention can be enhanced by encouraging clients to recognize and use their own internal capabilities to access resources and solve problems. The review also indicated that peer navigation, reducing structural, and system barriers, involving peers as major players in the health care team, and providing brief messages by healthcare providers enhance retention [27]. This is consistent with our intervention. We noted when retention improved, access to viral load monitoring and virologic suppression dramatically improved too. This confirms past recommendations of retaining HIV-positive persons in care in order to achieve viral load monitoring and suppression [1–4]. When HIV-positive adolescents remain in care, optimal care, treatment, and support are provided by the health system. This leads to better quality of care, virologic suppression, and reduced HIV morbidity and mortality.

### 4.1. Study Limitations

The findings in this QI intervention are consistent with the need to assess and improve retention of HIV-infected persons by strengthening and incorporating novel methods as well as by using casual analytical framework [31]. This study has demonstrated the critical role of QI in HIV care and treatment. It is important to recall that QI is a new intervention with scarce publications, and an overlooked approach for addressing gaps in HIV care. However, with simple, cost-effective, evidence-based, acceptable, and appropriate context-driven interventions like those demonstrated in this article, the quality of HIV/AIDS services and healthcare in general can be improved. Even though these interventions may be applicable to settings with similar challenges, several limitations should be considered. First, our intervention had fewer numbers of adolescents in a rural setting with relatively low mobility unlike in urban setting. Second, we did not establish the independent determinants of retention. Third, the absence of qualitative data to explain retention is another limitation. However, these limitations do not outweigh the design, conduct, and findings of the QI study. The figures presented constitute the total number of HIV-positive adolescents in care at the health facility. Besides, the interventions were rigorously planned, and were context specific and systematically implemented. Other than the uniqueness of the approach in improving healthcare performance in general, we have offered an insight in meeting the UNAIDS 90-90-90 targets through QI (in addition to other health sciences research approaches).

## 5. Conclusion and Recommendation

We have illustrated the contribution of QI in closing gaps in healthcare, specifically in HIV. We found retention of HIV-positive adolescents increased with tracking missed appointments, using mobile phone technology, starting an Adolescent Only Clinic, and holding monthly meetings to address HIV care and treatment challenges. We recommend the replication of these interventions in settings facing similar challenges in Uganda and elsewhere.

## Figures and Tables

**Table 1:** Theme matrix selection for prioritizing retention in HIV care according to age categories, Katooke HCIII, Kyenjojo district.

QI themes	Customers	A	B	A X B	Selected?
95.7% retention of children (below 10 years) in care	Patients	5	4	20	No
34.5% retention of HIV-positive adolescents (10–19 years) in care	Patients	5	5	25	Yes
87.3% retention of HIV-positive adults (20 years and above) in care	Patients	5	4.5	22.5	No

*Note*. A: impact score on patients; B: score on need to improve; AB: product of A and B; remarks: selected or not selected for QI.

## Data Availability

Data shall be shared on reasonable request to protect anonymity of participants.

## Abbreviations

AIDS: Acquired Immunodeficiency Syndrome
AOC: Adolescent Only Clinic
ART: Antiretroviral therapy
ARV: Antiretroviral drugs
CDC: Centers for Disease Control and Prevention
CME: Continuous medical education
HIV: Human immunodeficiency syndrome
PDSA: Plan-Do-Study-Act
PEPFAR: Presidential Emergency Plan for AIDS Relief
QI: Quality improvement
QIT: Quality improvement team.

## Supplementary Materials

Supplementary Materials Supplementary Material S1: viral load access and suppression levels versus retention.
