# Supplementary material for: Retention of HIV-Positive Adolescents in Care: A Quality Improvement Intervention in Mid-Western Uganda
Source: Biomed Res Int. 2018 May 6;2018:1524016. doi: 10.1155/2018/1524016 (PMC5960514; doi:10.1155/2018/1524016)
Supplement: Supplementary Materials — Supplementary Material S1: viral load access and suppression levels versus retention. [file 1524016.f1.pdf]

Supplementary material

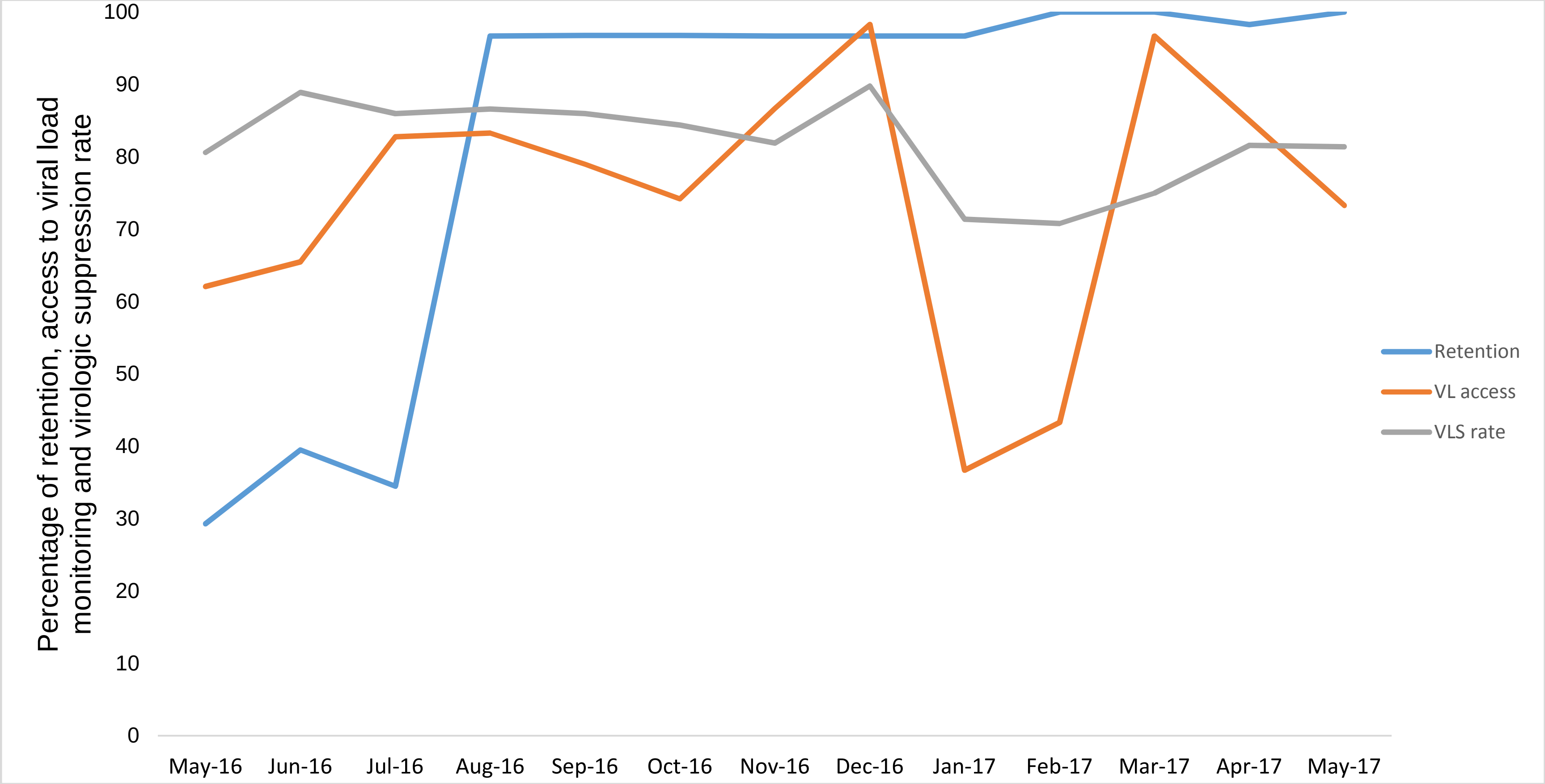

SUPPLEMENTARY MATERIAL S1: Viral load access and suppression levels versus retention

Note: VL: Viral load; VLS: Viral Load Suppression
